# Role of early extraction of odontogenic focus in deep neck infections

**DOI:** 10.4317/medoral.25536

**Published:** 2022-09-29

**Authors:** Jose Luis Treviño-Gonzalez, Karla M Santos-Santillana, Jose R Cortes-Ponce, Baltazar Gonzalez-Andrade, Josefina A Morales-del-Angel

**Affiliations:** 1 Otolaryngology and Head and Neck Surgery Division, School of Medicine and University Hospital “Dr. Jose E. González”, Universidad Autónoma de Nuevo León, Monterrey, Nuevo Leon, Mexico

## Abstract

**Background:**

Odontogenic deep neck infections remain a common condition that presents a challenging issue due to the complex involvement of the neck and adjacent structures and its potential life-threatening risk. Periapical infection of the second or third molar with spread to the submandibular and parapharyngeal spaces is the most commonly observed scenario. However, the time of dental extraction of the infection focus remains controversial. The aim of this study is to provide an overview of the epidemiology, clinical and radiological features, and management in patients diagnosed with ODNI and to identify the role of early dental extraction on patient outcomes and recovery.

**Material and Methods:**

This retrospective study included patients over 18 years old with a diagnosis of ODNI who were admitted to the University Hospital “Dr Jose Eleuterio Gonzalez” from January 2017 to January 2022. ODNI diagnosis was based on clinical and radiological evidence of the disease supplemented by dental and maxillofacial evaluation for an odontogenic aetiology.

**Results:**

A total of 68 patients were included in the study. The patients’ mean age was 40.96 ± 14.9. Diabetes mellitus was the most common comorbidity. The submandibular space was the most common deep neck space involved (n=59, 86.8%). Mediastinitis, marginal nerve injury and orocervical fistula were observed in 7.5% of patients, with no fatality in this series. A delay of >3 days for dental extraction of the involved tooth was associated with an increased rate of mediastinitis (n=3, 100%, *p*= 0.022), number of surgical interventions (1.45 ± 0.61, *p*= 0.006), ICU stay (n=8, 40%, *p*= 0.019), and ICU length of stay (0.85 ± 0.8, *p*= 0.001).

**Conclusions:**

Expedited management with surgical drainage and intravenous antibiotic treatment, along with early extraction of the involved tooth, is mandatory.

** Key words:**Odontogenic, neck infections, abscess, mediastinitis, tooth extraction.

## Introduction

Deep neck infections (DNI) remain a common condition that presents a challenging issue due to the complex involvement of the neck and adjacent structures and its potential life-threatening risk (1). Although its incidence has decreased with the use of antibiotics, DNI is a cause of substantial complications, including upper airway obstruction, mediastinitis, septic shock, and vascular thrombosis, leading to significant morbidity and mortality (2). Aetiology DNI has evolved with the antibiotic era, being pharyngotonsillitis the most common cause of DNI prior to antibiotic use (3). In the present day, dental infections are a persistent cause of DNI accounting for 33 to 65% of cases (4-6).

Odontogenic deep neck infections (ODNI) result mainly from periodontal diseases, periapical abscesses, and dental intervention in infected teeth. These infections have been considered an individual pathology from DNI, as ODNI display specific microbiological and treatment features (7,8). Periapical infection of the second or third molar with spread to the submandibular and parapharyngeal spaces is the most commonly observed scenario (9). However, a lack of identification of the origin of infection has been observed in 19.9% of cases (10). Despite the identification of a dental source of the infection, the time of dental extraction in ODNI remains controversial.

Regardless of the worldwide increase in dental hygiene and health campaigns, ODNI continues to be a cause of neck infections and fatal complications. The aim of our study is to provide an overview of the epidemiology, clinical and radiological features, and management in patients diagnosed with ODNI and to identify the role of early dental extraction on patient outcomes and recovery.

## Material and Methods

- Subjects

This retrospective study included patients over 18 years old with a diagnosis of ODNI who were admitted to the University Hospital “Dr Jose Eleuterio Gonzalez,” a tertiary referral academic hospital, from January 2017 to January 2022. Exclusion criteria included non-odontogenic DNI, pregnancy, superficial neck infections, surgical or penetrating infected neck wounds, incomplete medical records, and loss of follow-up after discharge. Patients with a history of neck surgery, head and neck cancer, radiotherapy, and chemotherapy were also excluded.

The research protocol was approved by the local Research and Institutional Ethics Committee. The authors assert that all procedures contributing to this work comply with the ethical standards of the relevant national and institutional guidelines on human experimentation and with the Helsinki Declaration of 1975, as revised in 2008.

- Data collection

Data were extracted from medical records using a standardized data collection form. All authors contributed to data retrieval and an independent author adjudicated any difference in interpretation between the data extractors.

- Studied variables

Studied variables included demographics, alcohol, Tabaco and drug use, symptomatology, radiological findings, treatment modality, microbiological cultures, outcomes, and complications. ODNI diagnosis was based on clinical and radiological evidence of the disease supplemented by dental and maxillofacial evaluation for an odontogenic aetiology. Radiological evaluation was performed by the assessment of computed tomographies obtained from the database of the University Hospital “Dr Jose E. González” Diagnostic Radiology Department. All scans were evaluated by head and neck radiologists and otolaryngologists.

- Statistical analysis

Statistical analysis was performed using SPSS V25.0 (Armonk, NY: IBM Corp). Categorical variables are reported as percentages and frequencies; continuous variables are reported as means and standard deviations. Categorical variables were compared using Pearson’s x2 test or Fisher’s exact test for 2 x 2 Tables. An unpaired Student’s t test or Mann–Whitney U test were used to compare continuous variables. *P*< 0.05 was considered statistically significant.

## Results

- Demographic data

A total of 111 patients with a diagnosis of DNI were extracted from medical records. Overall, 68 patients were included due to a confirmed ODNI diagnosis (61.3%). Main exclusion criteria included loss of follow-up (n= 10), head and neck cancer (n= 8), DNI associated with foreign body pharyngeal injury (n= 7), peritonsillar abscesses (n= 6), DNI associated with mandibular trauma, fractures, and cervical hematoma (n= 5), bacterial suppurative adenitis (n= 4), and necrotizing adenopathy associated with tuberculosis (n= 3). The patients’ mean age was 40.96 ± 14.9. A higher alcohol and tobacco use were observed in males when compared to females (n=8, 33.3% vs n=28, 63.6%; *p*= 0.017). Diabetes mellitus was the most common comorbidity, observed in 26.5% (n=18) of patients, followed by hypertension (n= 7, 10.3%), and HIV (n= 3, 4.4%). Odontalgia and cervical pain were the chief complaints in 89.7% and 86.8% of patients, respectively. Inferior molars were the most common affected teeth (n= 64, 94.1%) (Table 1). All patients were managed with incision and drainage after evaluation of the image scan.

**Table 1:**
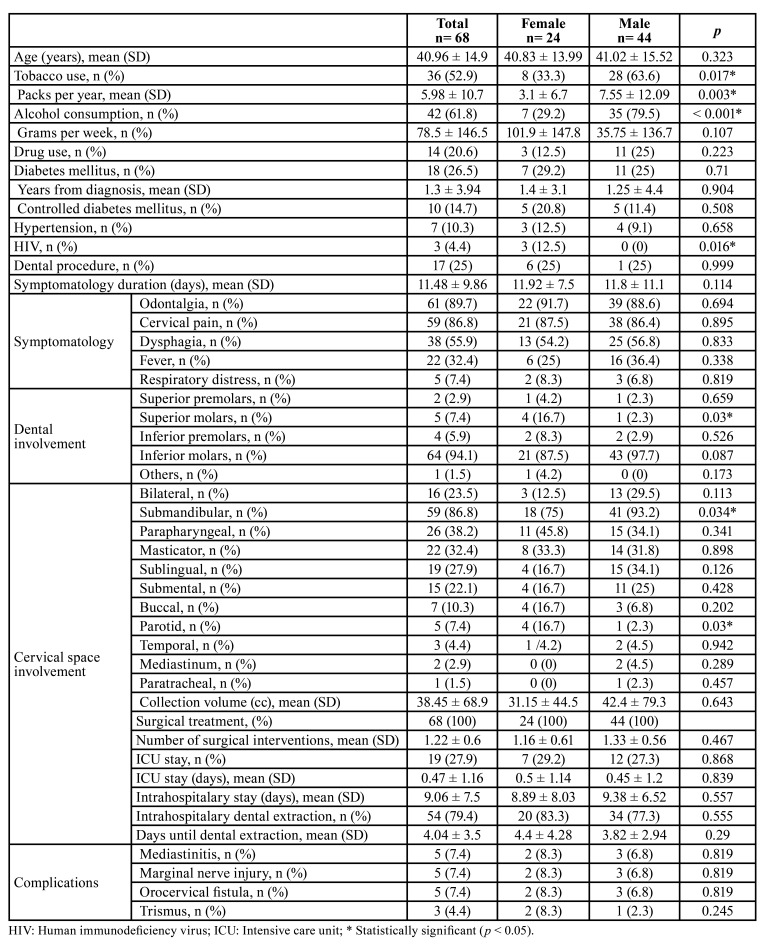
Patient demographics, clinical and radiological data, and outcomes.

- Clinical and radiological characteristics and patient outcomes

**Figure 1:**
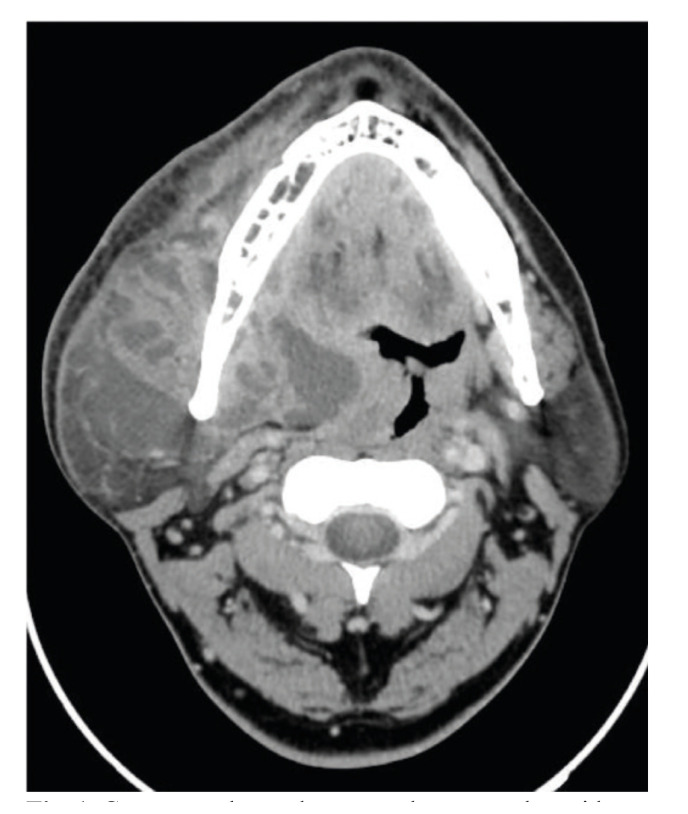
Contrast-enhanced computed tomography evidencing a multilocular abscess with extension to the submandibular, parapharyngeal, buccal, and masticator neck spaces.

**Figure 2:**
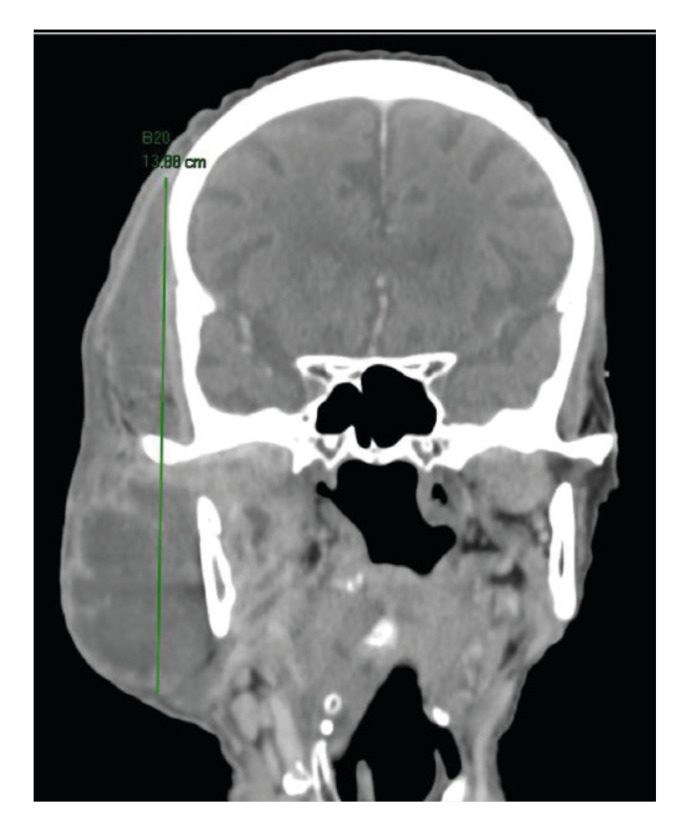
Contrast-enhanced computed tomography showing the presence of a deep neck abscess involving the buccal, masticator, parotid, and temporal spaces.

The submandibular space was the most common deep neck space involved (n=59, 86.8%) (Fig. 1), followed by the parapharyngeal (n=26, 38.2%), and the masticator space (n= 22, 32.4%) (Fig. 2), with a mean collection volume of 38.45 ± 68.9 cc. Bilateral involvement was reported in 25.5% (n=16) of cases.

Mediastinitis, marginal nerve injury and orocervical fistula were observed in 7.5% of patients, with no fatality in this series. Overall, in 79.4% (n= 54) of patients, intrahospitalary dental extraction was performed with a mean time until extraction of 4.04 ± 3.5 days (Table 1).

- Clinical characteristics and patient outcomes associated with involved dentition

Overall, inferior molars were the most commonly affected (n=64, 85.3%), with main involvement of submandibular (n=57, 83.8%), parapharyngeal (n=23, 33.8%), and masticator (n=21, 30.9%) neck spaces. Superior premolars and molars involvement were most frequently associated with ODNI of the parapharyngeal (n=7, 8.8%), submandibular (n= 5, 7.3%), and masticator space (n=3, 4.4%). ODNI of inferior molars had the highest rate of complications, without statistical significance (Table 2).

**Table 2:**
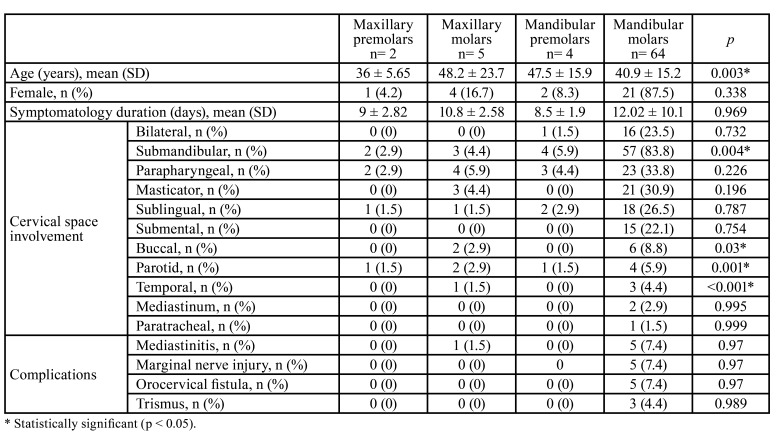
Clinical characteristics and patient outcomes associated with involved dentition.

A delay of >3 days for dental extraction of the involved tooth was associated with an increased rate of mediastinitis (n=3, 100%, *p* = 0.022), number of surgical interventions (1.45 ± 0.61, *p*= 0.006), ICU stay (n=8, 40%, *p* = 0.019), and ICU length of stay (0.85 ± 0.8, *p* = 0.001) (Table 3).

- Microbiological characteristics

Overall, 54 patients had a microbiological culture report. A negative culture was observed in 9 patients (11.5%). *Streptococcus* anginosus was the most commonly isolated microorganism (n=10, 14.7%), followed by *Streptococcus* constelatus (n=8, 11.8%), and *Staphylococcus* no aureus (n=6, 8.8%) (Table 4).

**Table 3:**
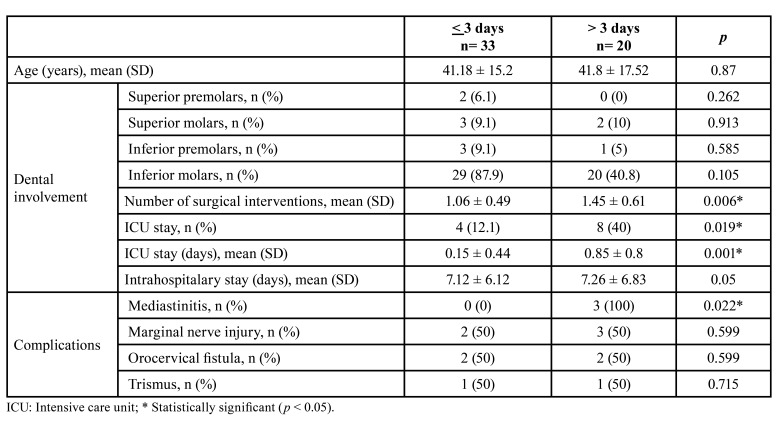
Involved dentition and patient outcomes associated with time to dental extraction.

**Table 4:**
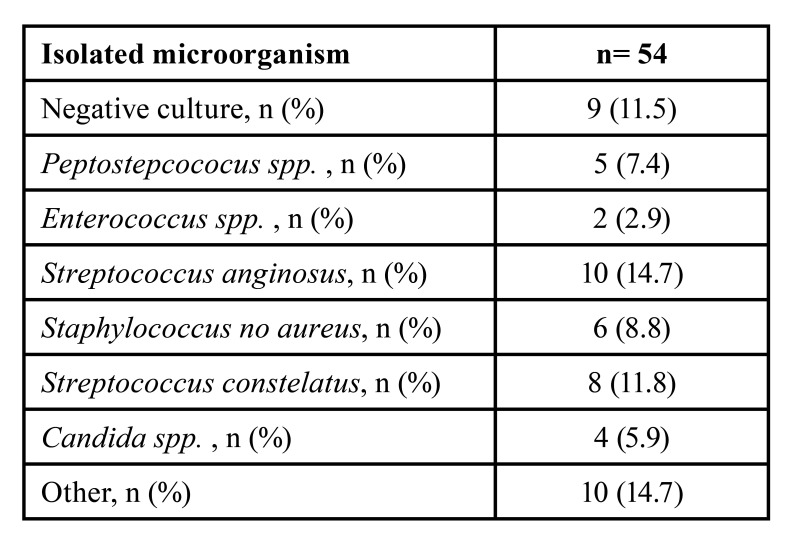
Microbiological characteristics.

## Discussion

In this observational study, we found that outcomes and complications of a patient with ODNI are associated with the involved dentation and the time of dental extraction since diagnosis. Inferior molars were the most common dentation involved in ODNI, with the most frequent extension to submandibular, parapharyngeal, and masticator cervical spaces. Furthermore, a delay of >3 days for dental extraction was associated with mediastinitis, ICU admission, and ICU length of stay. Overall, the complication rate was 26.6% in the present series.

Previous studies have assessed the prevalence, behaviour, and outcomes of ODNI. These infections usually result from pericoronitis and necrosis of the dental pulp with further root canal infection (11). The incidence of odontogenic infections as the leading cause of DNI varies among published evidence. However, odontogenic infections remain the principal cause of DNI (22 to 65.3%) (6,12,13). The second most common cause of DNI is debaTable, with IV drug use, trauma, and upper airway infections being the most common (14). However, unknown aetiology has been reported in up to 57% of cases (15). Patients with ODNI typically present with a history of dental pain or dental procedure, followed by cervical pain, fever, oedema, dysphagia, and trismus (14). Respiratory symptomatology, such as respiratory distress and stridor, is seen in more advanced stages of the disease.

Involved teeth have been associated with outcomes and prognosis in ODNI. Mandibular odontogenic infections have been associated with the need for surgical drainage, increased hospital stay, and admission to the ICU (14). In our series, mandibular molars were the most affected teeth, accounting for 55.3% of cases. Mandibular odontogenic infections tend to extend to submandibular, buccal, parotid, and temporal space than maxillary teeth and have a greater rate of complications.

Although infrequent, serious life-threatening complications can arise from ODNI, necrotizing fasciitis, sepsis, and mediastinitis being the most common. Dyspnea and dysphagia have been associated with the spread of ODNI toward deep cervical spaces and serve as an indicator of serious infections (2). ODNI was complicated by mediastinitis in 7.4% of cases in our study, similar to incidence reported in different series (2,6,16). Alotaibi *et al*. recommend admission in patients with signs of severity such as dyspnea, stridor, dysphagia, odynophagia, trismus, crepitus, and elevated white blood cell count (14). Specifically, a high C-reactive protein at admission has been associated with a more severe course of infection. It correlates with the length of hospital stay, the need for advanced airway management, more frequent ICU admissions, and complications (17,18).

The neutrophil to lymphocyte ratio (NLR) has recently shown promising results as a marker of infection and prognostic factor for various pathologies, including ODNI. Gallagher *et al*. correlated admission NLR with admission C-reactive protein and hospital length of stay, with a cut-off value for NLR of 4.65 for a length of stay > two days (19). Furthermore, Ban *et al*. proposed a cut-off value of 8.2 for NLR in predicting drainable collections in patients with DNI (20). However, cut-off values for NLR are still controversial, and an accurate value in ONDI is still missing.

Additional to serologic markers, length of stay has been significantly associated with the time of dental extraction. Removal of the infected focus is indispensable for recovery, and it has been widely recommended to extract the involved teeth without delay. However, the ideal time of removal is controversial. Despite the recommendation of primary teeth extraction simultaneously with surgical drainage and intravenous antibiotics, odontogenic focuses are frequently extracted at a secondary time, mainly due to a limited mouth aperture (11). Heim *et al*. observed the lowest length of stay in patients treated with surgical drainage and extraction simultaneously (11). Velhonoja *et al*. observed a reduction of 2 days of intrahospitalary stay when dental extraction was performed simultaneously with the incision and drainage. No differences between complications and ICU stay were observed (15). Conversely, in our study, we observed a decrease in the number of patients admitted to the ICU, lower ICU length of stay, and mediastinitis in patients with dental extraction performed in the first three days of admission. Treviño *et al*. observed that a delayed time for dental extraction was associated with a length of hospital stay of >7 days (6).

Regardless of the time of dental extraction, early management of ODNI with surgical drainage and intravenous antibiotic therapy is imperative. Specifically, gas formation identified in computed tomography has been associated with a higher complication rate, increased mortality, need for reintervention, and prolonged hospital and ICU stay (15). Reintervention rate among our population is 8%, which is associated with diabetes mellitus, the involvement of masticator and temporal space, and delayed dental extraction (6). With this in mind, expedited surgical and antibiotic management in these patients is crucial to avoid the infection extension, rates of complications and mortality, and the need for multiple surgical interventions.

Limitations of this study are mostly due to its retrospective design. Additionally, ICU stay and hemodynamical instability in patients with advanced ODNI could have delayed dental extraction.

ODNI remain a common condition with potentially life-threatening complications. Expedited management with surgical drainage and intravenous antibiotic treatment, along with the extraction of the involved tooth is mandatory. In our study, we observed a reduced number of surgical interventions, complications, and a decreased ICU length of stay in patients with early extraction of the dental infection focus. Larger, prospective studies are needed to support our findings.

## References

[1] Wang LF, Kuo WR, Tsai SM, Huang KJ (2003). Characterizations of life-threatening deep cervical space infections: a review of one hundred ninety-six cases. Am J Otolaryngol.

[2] Suebara AB, Goncalves AJ, Alcadipani FA, Kavabata NK, Menezes MB (2008). Deep neck infection-analysis of 80 cases. Rev Bras Otorrinolaringol.

[3] Almutairi DM, Alqahtani RM, Alshareef N, Alghamdi YS, Al-Hakami HA, Algarni M (2020). Deep Neck Space Infections: A Retrospective Study of 183 Cases at a Tertiary Hospital. Cureus.

[4] Chi TH, Tsao YH, Yuan CH (2014). Influences of patient age on deep neck infection: clinical etiology and treatment outcome. Otolaryngol Head Neck Surg.

[5] Parhiscar A, Har-El G (2001). Deep neck abscess: a retrospective review of 210 cases. Ann Otol Rhinol Laryngol.

[6] Treviño-Gonzalez JL, Maldonado-Chapa F, González-Larios A, Morales-del-Angel JA, Soto-Galindo GA, Zafiro Garcia- Villanueva JM (2021). Deep Neck Infections: Demographic and Clinical Factors Associated with Poor Outcomes. ORL J Otorhinolaryngol Relat Spec.

[7] Kinzer S, Pfeiffer J, Becker S, Ridder GJ (2009). Severe deep neck space infections and mediastinitis of odontogenic origin: clinical relevance and implications for diagnosis and treatment. Acta Otolaryngol.

[8] Ridder GJ, Technau-Ihling K, Sander A, Boedeker CC (2005). Spectrum and management of deep neck space infections: an 8-year experience of 234 cases. Otolaryngol Head Neck Surg.

[9] Bottin R, Marioni G, Rinaldi R, Boninsegna M, Salvadori L, Staffieri A (2003). Deep neck infection: a present-day complication. A retrospective review of 83 cases (1998-2001). Eur Arch Otorhinolaryngol.

[10] Marioni G, Staffieri A, Parisi S, Marchese-Ragona R, Zuccon A, Staffieri C (2010). Rational diagnostic and therapeutic management of deep neck infections: analysis of 233 consecutive cases. Ann Otol Rhinol Laryngol.

[11] Heim N, Warwas FB, Wiedemeyer V, Wilms CT, Reich RH, Martini M (2019). The role of immediate versus secondary removal of the odontogenic focus in treatment of deep head and neck space infections. A retrospective analysis of 248 patients. Clin Oral Investig.

[12] Kataria G, Saxena A, Bhagat S, Singh B, Kaur M, Kaur G (2015). Deep Neck Space Infections: A Study of 76 Cases. Iran J Otorhinolaryngol.

[13] Boscolo-Rizzo P, Marchiori C, Montolli F, Vaglia A, Da Mosto MC (2006). Deep neck infections: a constant challenge. ORL J Otorhinolaryngol Relat Spec.

[14] Alotaibi N, Cloutier L, Khaldoun E, Bois E, Chirat M, Salvan D (2015). Criteria for admission of odontogenic infections at high risk of deep neck space infection. Eur Ann Otorhinolaryngol Head Neck Dis.

[15] Velhonoja J, Lääveri M, Soukka T, Irjala H, Kinnunen I (2020). Deep neck space infections: an upward trend and changing characteristics. Eur Arch Otorhinolaryngol.

[16] Yang W, Hu L, Wang Z, Nie G, Li X, Lin D (2015). Deep neck infection: a review of 130 cases in southern China. Medicine.

[17] Ylijoki S, Suuronen R, Jousimies-Somer H, Meurman JH, Lindqvist C (2001). Differences between patients with or without the need for intensive care due to severe odontogenic infections. J Oral Maxillofac Surg.

[18] Riekert M, Kreppel M, Zöller JE, Ziek M, Annecke T, Schick VC (2019). Severe odontogenic deep neck space infections: risk factors for difficult airways and ICU admissions. Oral Maxillofac Surg.

[19] Gallagher N, Collyer J, Bowe CM (2021). Neutrophil to lymphocyte ratio as a prognostic marker of deep neck space infections secondary to odontogenic infection. Br J Oral Maxillofac Surg.

[20] Ban MJ, Jung JY, Kim JW, Park KN, Lee SW, Koh YW (2018). A clinical prediction score to determine surgical drainage of deep neck infection: A retrospective case-control study. Int J Surg.

